# Flight Performance of *Mamestra brassicae* (Lepidoptera: Noctuidae) Under Different Biotic and Abiotic Conditions

**DOI:** 10.1093/jisesa/iez126

**Published:** 2020-01-03

**Authors:** Jiang-Long Guo, Xiao-Kang Li, Xiu-Jing Shen, Meng-Lun Wang, Kong-Ming Wu

**Affiliations:** 1 College of Plant Protection, Shenyang Agricultural University, Shenyang, China; 2 State Key Laboratory for Biology of Plant Diseases and Insect Pests, Institute of Plant Protection, Chinese Academy of Agricultural Sciences, Beijing, PR China

**Keywords:** *Mamestra brassicae*, flight mill, flight ability, stroboscope, wingbeat frequency

## Abstract

*Mamestra brassicae* L. is an important, regionally migratory pest of vegetable crops in Europe and Asia. Its migratory activity contributes significantly to population outbreaks, causing severe crop yield losses. Because an in-depth understanding of flight performance is key to revealing migratory patterns, here we used a computer-linked flight mill and stroboscope to study the flight ability and wingbeat frequency (WBF) of *M. brassicae* in relation to sex, age, temperature, and relative humidity (RH). The results showed that age significantly affected the flight ability and WBF of *M. brassicae*, and 3-d-old individuals performed the strongest performance (total flight distance: 45.6 ± 2.5 km; total flight duration: 9.3 ± 0.3 h; WBF: 44.0 ± 0.5 Hz at 24°C and 75% RH). The age for optimal flight was considered to be 2–3 d old. Temperature and RH also significantly affected flight ability and WBF; flight was optimal from 23°C to 25°C and 64–75% RH. Because *M. brassicae* thus has great potential to undertake long-distance migration, better knowledge of its flight behavior and migration will help establish a pest forecasting and early-warning system.

Migration, as an adaptive life-history strategy for survival and reproduction, allows insects to avoid adverse environmental conditions and exploit temporary or patchy habitats, promoting population spread and gene flow (Dingle and Drake 2007, Chapman et al. 2011, Nagoshi et al. 2017, Cao and Wu 2019). Every year, numerous insects belonging to various taxonomic groups (e.g., Lepidoptera, Orthoptera, Hemiptera) migrate aerially by day or night on a continental scale (Chapman and Drake 2010, Hobson et al. 2012, Chapman et al. 2015), which has a major impact on ecosystem function (Bauer and Hoye 2014). The migration of lepidopteran agricultural pests such as *Mythimna separata* (Walker), *Spodoptera exigua* (Hübner), *Spodoptera frugiperda* (J.E.Smith), is especially important and has led to severe yield losses (Jiang et al. 2011, Zheng et al. 2011, Stokstad 2017). Because the frequent use of insecticides to control these pests has negative side effects such as insect resistance and toxic residues (Cartea et al. 2014, Calvo-Agudo et al. 2019), increasing our knowledge of pest migration is of particular importance for forecasting and early-warning systems and integrated pest management (IPM).

Flight mills, tools that can keep a variety of insects flying continuously, are convenient for estimating insect relative migratory performance in the laboratory (Taylor et al. 2010, Liu et al. 2011, Ávalos et al. 2014, Attisano et al. 2015) and measuring flight variables (e.g., distance, duration, speed) within a set period and the effects of age, sex, or temperature on an insect’s flight ability (Attisano et al. 2015; Fu et al. 2017a,b; Minter et al. 2018). Since 1950s, tethered flight mills have been extensively applied to evaluate flight performance of multiple migratory moth species, such as *Agrotis ipsilon* (Hufnagel) (Sappington and Showers 1991), *Helicoverpa armigera* (Hübner) (Armes and Cooter 1991), *Spodoptera litura* (Fabricius) (Murata and Tojo 2004), *S. exigua* (Han et al. 2008), and *Ctenoplusia agnata* (Staudinger) (Fu et al. 2017b).

Wingbeat frequency is another major variable that is related to the aerodynamic analyses of insect flight (Altshuler et al. 2005). Because insects depend on wing beats to maintain flight during migration (e.g., ascending to cruise altitude) (Huang et al. 2013), wingbeat frequency (WBF) may prove useful for evaluating relative flight performance of an insect.

*Mamestra brassicae* L. is an important pest of vegetable crops and mainly distributed in a geographic belt from 30°N to about 70°N in Europe and Asia (Turnock and Carl 1995, Shi et al. 2005, Wu et al. 2015). In China, *M. brassicae* usually undergoes one to four generations each year depending on the latitude, e.g., it may have three to four generations in Chongqing (28°N-32°N) and two generations in Heilongjiang Province (43°N-53°N) (Wu et al. 2015). This pest is mainly present from spring to autumn (May to October) and overwinters as pupa in the soil. *Mamestra brassicae* larvae are polyphagous (of more than 70 host plant species of 22 families), preferring Brassicaceae and Chenopodiaceae (Popova 1993, Rojas et al. 2000) and damaging leaves by feeding (Cartea et al. 2014). By using a searchlight trap to monitor migratory insects on a small island, in the center of the Bohai Strait in northern China, Wu et al. (2015) for the first time confirmed that *M. brassicae* is a migratory species and annually migrates over long distances in northeastern China. Its migration behavior contributes significantly to intermittent population outbreaks, which cause severe damage and yield losses of vegetables each year and makes timely control of this pest difficult (Wu et al. 2015). Migratory insects usually are strong fliers, and an in-depth understanding of flight behavior is critical to revealing their migratory pattern (Sappington and Showers 1991, Fu et al. 2017a,b).

We thus used computer-linked flight mills and a stroboscope to study the effect of biotic (age, sex) and abiotic factors (temperature, relative humidity) on the flight ability and WBF of *M. brassicae*. This work further strengthens our understanding of the flight and migration behavior of *M. brassicae* and will contribute to the timely development of an effective regional forecasting and management protocol.

## Materials and Methods

### Insect Culture

*Mamestra brassicae* moths were collected from April to May at Beihuang Island, Shandong Province, China (38°23′N, 120°55′E) and used to establish a laboratory culture in an artificial climate incubator at 24 ± 1°C, 75 ± 5% relative humidity (RH), and 16 h:8 h (L: D) photoperiod. First to fifth instar larvae were reared on an artificial diet in a plastic petri dish (high × diameter = 2.5 × 10 cm), then mature larvae were transferred to a plastic box (length × width × height = 20.5 × 15.5 × 8.5 cm) filled with sterile soil until pupation. Pupae were removed from the soil and sexed (Zhao et al. 2011). To determine the age of unmated moths, male and female pupae were placed separately in plastic buckets (height × diameter = 15 × 11 cm) and checked daily for emergence. Moths were provided with 5% (vol: vol) honey solution via cotton wicks as supplemental food every day.

### Flight Mill and Tethered Flight

Flight variables of the moths were tested using a 24-channel, tethered flight system (Jiaduo Industry & Trade Co., Ltd, Hebi, China), which mainly consisted of a computer, flight mills, and acquisition system, similar to the apparatus described by Beerwinkle et al. (1995). Before the test, each moth specimen was lightly anesthetized for 10–20 s in a glass bottle with a cotton wick soaked with ethyl acetate at the bottom. The moths were then attached by the dorsum at the junction between the metathorax and abdomen to the end of the flight mill arm (length × diameter = 30 × 0.07 cm), with 502 quick-drying glue (M&G Chenguang Stationery Co., Ltd, Shanghai, China). The other end of the arm was wrapped in tin foil until the mill was balanced. The flight mill was installed in a completely dark room. The temperature and humidity inside the room could be adjusted to meet test requirements.

The time of flight initiation and cessation and the number of mill revolutions every 5 s were recorded. Any flight bout that stopped for >1 min was considered to have ended. Based on these raw data, various variables such as total flight distance and duration could be calculated to characterize the flight potential of *M. brassicae*. Because a single variable, however, might fail to reveal flight differences between different treatments in insects or produce misleading results (Luo et al. 2002), we evaluated relative flight ability of *M. brassicae* based on six variables: total flight distance, total flight duration, mean flight speed, longest distance of one flight, longest duration of one flight, and mean flight bouts. The tethered-flight tests were started at 8:00 pm and finished at 8:00 am.

### Measuring WBF

A stroboscope (Phaser-Strobe pbx, Monarch) and the method of Huang et al. (2013) was used to measure the WBF after the moth attached to the flight mill arm had acclimatized for 10 min in the test conditions in the climatic chamber. The frequency of the flashing light was adjusted from high to low. When the moth’s wings remained visually static, the data displayed on the screen of the apparatus were the WBF of moths.

### Experimental Design

In the first of three assays, unmated *M. brassicae* males and females of different ages (1, 3, 5, 7, and 9 d old) were tested for the effect of sex and age on flight ability and WBF at 24°C and 75% RH. In the second assay, according to findings obtained from the first assay, 3-d-old unmated *M. brassicae* males and females were tested for the effect of temperature (12, 16, 20, 24, 28, and 32°C) on flight ability and WBF at 75% RH. In the third assay, 3-d-old unmated *M. brassicae* males and females were tested for the effect of RH (30, 45, 60, 75, 90, and 100%) on flight ability and WBF at 24°C. For each treatment, moths that died or escaped during the test were excluded, and a minimum of 25 individuals were tested.

### Data Analyses

Differences in flight ability and WBF between males and females of a given age were tested for significance using Student’s *t*-test. The effect of age, temperature, and RH on flight ability and WBF of *M. brassicae* was analyzed for significant differences using a one-way ANOVA followed by Tukey’s honestly significant difference (HSD) test. To check assumptions of normality and homogeneity for parametric analysis, the Kolmogorov–Smirnov test and Levene’s test were used before analyses, and if the assumptions were not met, flight data were log-transformed or ranked. To find the optimum age, temperature or RH for flight, a nonlinear model (Gaussian function) $y=\frac{D}{B\sqrt{2\pi }}e^{-\frac{{\left(x-A\right)}^{2}}{2B^{2}}}$ or $y=S-\frac{D}{B\sqrt{2\pi }}e^{-\frac{{\left(x-A\right)}^{2}}{2B^{2}}}$was used to describe the relationship between flight variables (e.g., total flight distance, mean flight speed) and key variables: age, temperature and RH. In the model, *y* represents a flight variable, *x* represents a key variable, and S, D, A, and B are constants. Because WBF did not vary distinctly in relation to the above variables, WBF data were excluded from the nonlinear regression analysis. All data procedures were executed with SPSS 20.0 (SPSS Inc., Chicago, IL).

## Results

### Effect of Sex and Age on Flight Ability and WBF

**Fig. 1.**
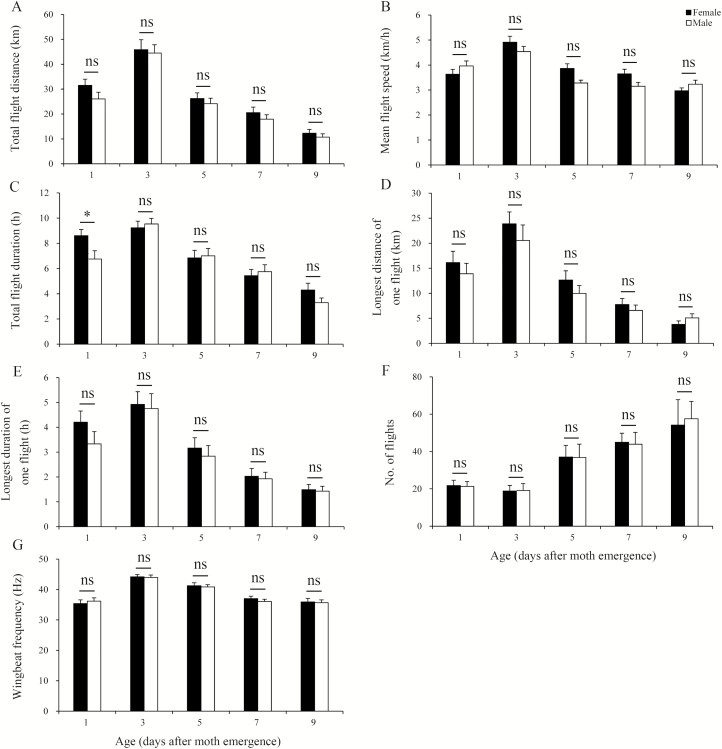
Mean (±SE) flight performance variables for 1- to 9-d-old male and female *M. brassicae* at 24°C and 75% RH. An asterisk above the bars indicates a significant difference between males and females; ns indicates no significant difference was found between males and females at the 5% level of significance in Student’s *t*-test. (A) Total flight distance. (B) Mean flight speed. (C) Total flight duration. (D) Longest distance of one flight. (E) Longest duration of one flight. (F) No. of flight. (G) Wingbeat frequency.

Using the results of the 12-h tethered flight and WBF experiments, we compared flight performance between *M. brassicae* males and females. Except for the total flight duration in 1-d-old individuals, there were no significant differences in flight variables between males and females at any given age (Fig. 1).

**Fig. 2.**
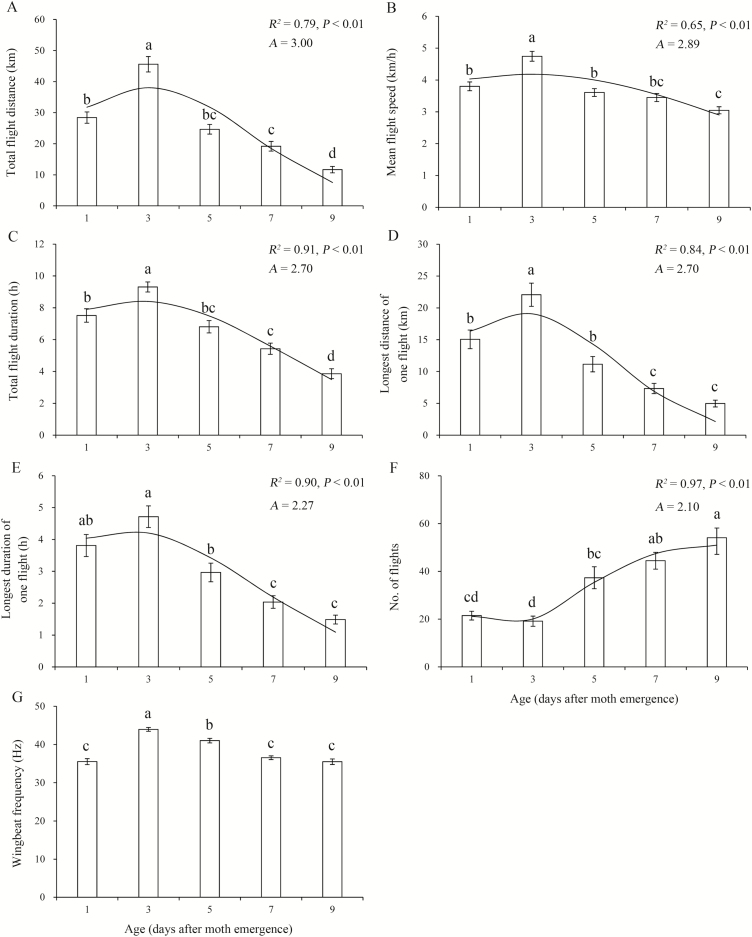
Mean (±SE) flight performance variables as a function of age for *M. brassicae* at 24°C and 75% RH. Bars sharing the same letter indicate that there are no significant differences at the 5% level in Tukey’s HSD tests. The curve represents the relationship between the variable and adult age. *R*^2^ and *P* for the nonlinear model are shown above the curve for each variable; *A* = optimal age for flight predicted by the model. (A) Total flight distance. (B) Mean flight speed. (C) Total flight duration. (D) Longest distance of one flight. (E) Longest duration of one flight. (F) No. of flight. (G) Wingbeat frequency.

Age significantly affected all flight variables (Table 1). With the exception of the number of flight bouts (which tended to increase with aging), flight variables (e.g., total flight distance, duration, WBF) first increased and then decreased from 1 to 9 d of age, with the highest values in 3-d-old individuals (e.g., total flight distance: 45.6 ± 2.5 km; total flight duration: 9.3 ± 0.3; WBF: 44.0 ± 0.5 Hz at 24°C and 75% RH) (Fig. 2). Furthermore, the number of flight bouts was lower in 1 to 3 d old than in 5 to 9 d old (Fig. 2F). The fitted nonlinear functions showed that maximum values for total flight distance, mean flight speed, total flight duration, longest distance of one flight, and longest duration of one flight were found at ages of 3.0, 2.9, 2.7, 2.7, and 2.3 d, respectively, and the fewest flight bouts were found for age of 2.1 d (Fig. 2A–F).

**Table 1.** One-way ANOVA of flight performance variables of *M. brassicae* as a function of age

Source	df	Total flight distance		Mean flight speed		Total flight duration		Longest distance of one flight		Longest duration of one flight		No. of flights		WBF	
		*F*	*P*	*F*	*P*	*F*	*P*	*F*	*P*	*F*	*P*	*F*	*P*	*F*	*P*
Age	4	43.83	<0.01	23.49	<0.01	33.59	<0.01	32.23	<0.01	26.10	<0.01	18.80	<0.01	38.61	<0.01
Error	268														
Total	272														

### Effect of Temperature on Flight Ability and WBF

**Fig. 3.**
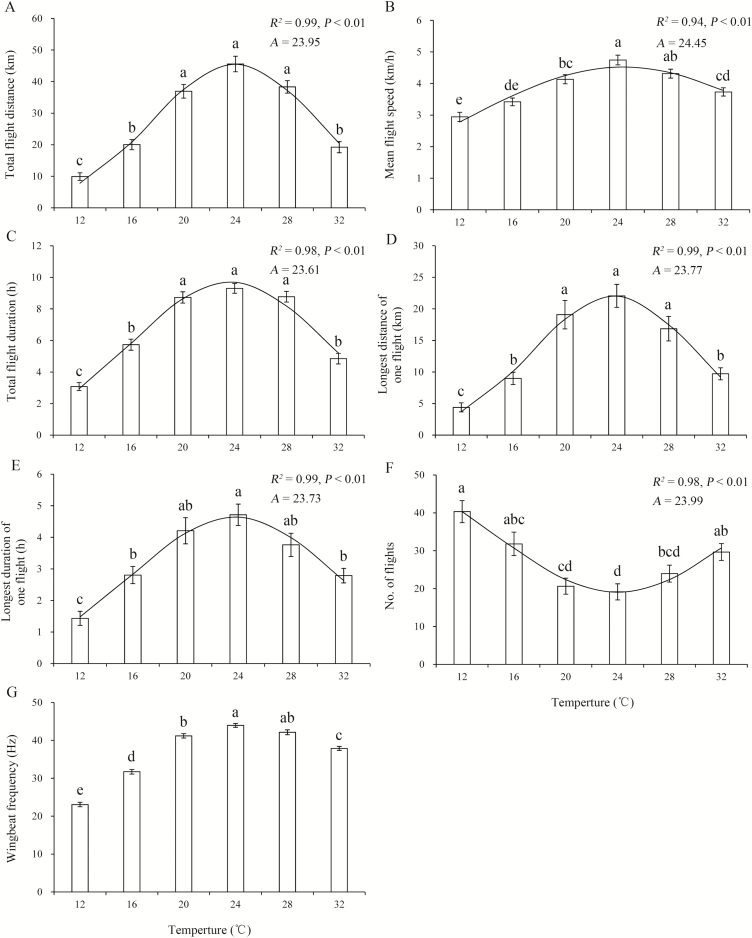
Means ± SE for flight performance as a function of temperature for 3-d-old *M. brassicae* at 75% RH. Bars sharing the same letter indicate that there are no significant differences at the 5% level in Tukey’s HSD tests. The curve represents the relationship between the variable and adult temperature. *R*^2^ and *P* for the nonlinear model are shown above the curve for each variable; *A* = optimal temperature for flight predicted by the model. (A) Total flight distance. (B) Mean flight speed. (C) Total flight duration. (D) Longest distance of one flight. (E) Longest duration of one flight. (F) No. of flight. (G) Wingbeat frequency.

For 3-d-old *M. brassicae* moths at 75% RH, the one-way ANOVA revealed that temperature significantly affected all flight variables (Table 2). As temperature increased, for all flight variables except the number of flight bouts, values first increased and then decreased, and values were higher at 20–28°C than at 12, 16, or 32°C, with the highest values at 24°C (Fig. 3A–E,G). However, there were fewer flight bouts at 20–28°C than at the other temperatures (Fig. 3F). The fitted nonlinear functions showed that maximum values for total flight distance, mean flight speed, total flight duration, longest distance of one flight, and longest duration of one flight were found at temperatures of 24.0, 24.4, 23.6, 23.8, and 23.7°C, respectively, and the fewest flight bouts occurred at 24.0°C (Fig. 3A–F).

**Table 2.** One-way ANOVA of flight performance variables of *M. brassicae* as a function of temperature

Source	df	Total flight distance		Mean flight speed		Total flight duration		Longest distance of one flight		Longest duration of one flight		No. of flights		WBF	
		*F*	*P*	*F*	*P*	*F*	*P*	*F*	*P*	*F*	*P*	*F*	*P*	*F*	*P*
Temperature	5	59.67	<0.01	21.62	<0.01	60.12	<0.01	30.24	<0.01	19.74	<0.01	10.30	<0.01	188.91	<0.01
Error	323														
Total	328														

### Effect of RH on Flight Ability and WBF

**Fig. 4.**
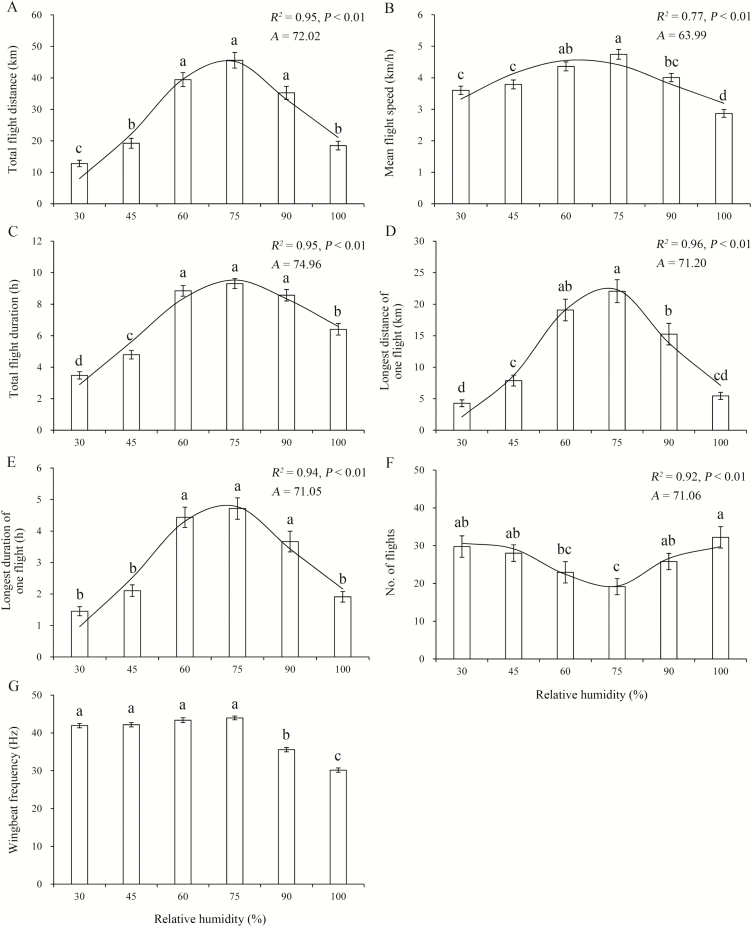
Means ± SE for flight performance as a function of RH for 3-d-old *M. brassicae* at 24°C. Bars sharing the same letter indicate that there are no significant differences at the 5% level by Tukey’s HSD tests. The curve represents the relationship between the variable and RH. *R*^2^ and *P* for the nonlinear model are shown above the curve for each variable; *A* = optimal RH for flight predicted by the model. (A) Total flight distance. (B) Mean flight speed. (C) Total flight duration. (D) Longest distance of one flight. (E) Longest duration of one flight. (F) No. of flight. (G) Wingbeat frequency.

For 3-d-old moths at 24°C, the one-way ANOVA revealed that RH significantly affected all flight variables (Table 3). More specifically, as the RH increased, values for all parameters except for the number of flight bouts first increased and then decreased. *M. brassicae* moths had relatively higher values for flight variables at 60–75% RH than at other RH levels, with the highest values at 75% RH (Fig. 4A–E). The value for the number of flight bouts was fewest at 75% RH (Fig. 4F). Furthermore, the WBF was higher at relatively low RH (30–75%) than at high RH (90–100%) (Fig. 4G). The fitted nonlinear functions showed that maximum values for total flight distance, mean flight speed, total flight duration, longest distance of one flight, and longest duration of one flight were found at RH 72.0, 64.0, 75.0, 71.2, and 71.1%, respectively, and the fewest flight bouts occurred at 71.1% RH (Fig. 4A–F).

**Table 3.** One-way ANOVA analysis of flight performance variables of *M. brassicae* as a function of RH

Source	df	Total flight distance		Mean flight speed		Total flight duration		Longest distance of one flight		Longest duration of one flight		No. of flights		WBF	
		*F*	*P*	*F*	*P*	*F*	*P*	*F*	*P*	*F*	*P*	*F*	*P*	*F*	*P*
RH	5	47.74	<0.01	22.31	<0.01	57.61	<0.01	43.07	<0.01	34.52	<0.01	5.35	<0.01	93.10	<0.01
Error	332														
Total	337														

## Discussion

Many empirical studies of insects have shown that flight performance is influenced by various biotic and abiotic factors (Bowler and Benton 2005; Sarvary et al. 2008; Huang et al. 2013; Fu et al. 2017a,b; Villarreal et al. 2017; Minter et al. 2018; Qin et al. 2018). Without exception, we similarly found in this study that age, temperature, and RH significantly affected flight ability and WBF of *M. brassicae*.

To date, migratory noctuids such as *A. ipsilon* (Sappington and Showers 1991), *M. separata* (Luo et al. 1999), *S. litura* (Tu et al. 2010), *Macdunnoughia crassisigna* Warren (Fu et al. 2017a), and *C. agnata* (Fu et al. 2017b) have shown strong flight ability in tethered flight experiments. Due to the inherent limitations of tethered flight, however, results obtained from flight mills cannot reflect flight performance of a given insect species in natural conditions (Armes and Cooter 1991). However, relative flight performance can reasonably be evaluated using flight mills (Taylor et al. 2010). In our study, 3-d-old individuals of *M. brassicae* flew, respectively, a mean distance of 45.6 ± 2.5 km, with a mean speed of 4.8 ± 0.2 km/h in a 12-h assay. The flight performance of *M. brassicae* is similar to that of other migratory insects such as *H. armigera* (Wu and Guo 1996), *S. litura* (Tu et al. 2010), and was stronger than that of *Mythimna loreyi* (Qin et al. 2018). In addition, during a 12-yr monitoring period, >30,000 *M. brassicae* specimens were captured on Beihuang Island, demonstrating that this species migrates across the Bohai Strait (Wu et al. 2015). Therefore, all these results suggest that *M. brassicae* has strong potential for long-distance flight.

Age-dependent variation in flight performance has been documented for a wide range of insect species, such as *H. armigera* (Coombs 1997, Shi et al. 2013), *Adelphocoris suturalis* Jakovlew (Lu et al. 2009), *Cnaphalocrocis medinalis* (Guenée) (Huang et al. 2013), *C. agnata* (Fu et al. 2017b), *Mythimna loreyi* Walker (Qin et al. 2018). Generally speaking, the general trend is that flight performance of a migratory insect increases gradually after adult emergence and peaks at a relatively early stage of adult life and then declines with age (Dingle 1985, Coombs 1997). In the present study, both sexes of *M. brassicae* also showed similar age-related changes in flight performance, with the strongest flight in 3-d olds. The variation in flight performance may be caused by internal species-specific temporal changes in physiology (Farnworth 1972, Saito 2000). Insect flight is closely related to the development of flight muscles (Heinrich 1971, Marden 2000, Cheng et al. 2016). Flight performance likely increases in early adults owing to increased mass of the flight muscles (Lorenz 2007). However, as age continues to increase, juvenile hormone in the hemolymph induces or accelerates flight muscle histolysis (Borden and Slater 1968, Shiga et al. 2002), which diminishes flight performance. This flight muscle histolysis has been confirmed in insects such as *M. separata* (Luo 1996), *Gryllus bimaculatus* (Lorenz 2007), and *Pieris napi* (Stjernholm and Karlsson 2008). Given that *M. brassicae* moths had a short prereproductive phase and the flight ability of 3-d-old mated moths was lower than that of 3-d-old unmated moths (Wu et al. 2016), we speculate that migratory flights may occur at least before insects are 3 d old. Although this assumed time window for optimal migration for *M. brassicae* seems narrow, this species, like other migratory insects, might use fast-moving winds for rapid, comparatively long-distance migration (Chapman et al. 2016, Hu et al. 2016).

As poikilotherms, migratory insects and their flight performance can be affected by many environmental factors, especially temperature and humidity (Kroder et al. 2006, Huang et al. 2013, Minter et al. 2018). Large-scale insect migration usually occurs at a suitable temperature and humidity (Reynolds et al. 2005, Wood et al. 2009), and extreme conditions (e.g., very high temperature, low humidity) can inhibit flight performance (Liu et al. 2011, Huang et al. 2013, Fu et al. 2017a,b) as it did in our study. The optimum temperature was 23–25°C, and optimum RH was 64–75% for flight which is similar to the results for other noctuid insects such as *H. armigera* (Armes and Cooter 1991, Shi et al. 2013), *S. litura* (Tu et al. 2010), *M. crassisigna* (Fu et al. 2017a), and *C. agnata* (Fu et al. 2017b). The main reasons that unfavorable conditions (e.g., high temperature, low humidity) inhibit flight performance may be due to water loss (Farnworth 1972; Lu et al. 2007; Fu et al. 2017a,b), or because moths flying at high ambient temperature cannot dissipate the heat generated by wing muscles rapidly enough, leading to heat stress (Unwin and Corbet 1984). These findings are important for understanding the migration pattern of *M. brassicae*. For example, by using suitable temperature and humidity range for flight, we can estimate the flight altitude of migratory *M. brassicae* (many noctuids are known to fly in altitudinal layers; Feng et al. 2003, 2009; Reynolds et al. 2005; Wood et al. 2009). Knowledge of the optimal flight attitude will allow more accurate estimates of migration trajectory.

In summary, the flight mill and stroboscope, as fairly easy-to-use tools, can provide quantitative estimates of the relative flight performance of *M. brassicae* under various conditions. These findings can help us understand the migration of *M. brassicae* and develop effective forecasting and management programs. However, as mentioned above, tethered flight has limitations (Armes and Cooter 1991, Beerwinkle et al. 1995, Taylor et al. 2010, Minter et al. 2018), and caution is needed when extrapolating results from laboratory flight-mill experiments. For insights into the full migratory cycle of *M. brassicae*, future studies should integrate the flight mill and stroboscope with other techniques such as field sampling and entomological radar, trajectory, and population genetics analyses to thoroughly research the migratory behavior of this species (Minter et al. 2018).
